# Physicians’ attitudes toward unhealthy alcohol use and self-efficacy for screening and counseling as predictors of their counseling and primary care patients’ drinking outcomes

**DOI:** 10.1186/1747-597X-8-17

**Published:** 2013-05-30

**Authors:** A Rani Elwy, Nicholas J Horton, Richard Saitz

**Affiliations:** 1 Center for Health Quality, Outcomes and Economic Research, Edith Nourse Rogers Memorial VA Hospital, Department of Veterans Affairs, 200 Springs Road (152), Bedford, MA 01730, USA; 2 Department of Health Policy and Management, Boston University School of Public Health, 715 Albany Street, Talbot 3W, Boston, MA 02118, USA; 3 Clinical Addiction Research and Education Unit, Section of General Internal Medicine, Boston University School of Medicine, 801 Massachusetts Avenue, 2nd Floor, Boston, MA 02118, USA; 4 Department of Mathematics, Amherst College, Box 2239, PO 5000, Amherst, MA 01002, USA

**Keywords:** Self-efficacy, Attitudes, Screening, Counseling, Physicians, Unhealthy alcohol use

## Abstract

**Objective:**

Patients’ unhealthy alcohol use is often undetected in primary care. Our objective was to examine whether physicians’ attitudes and their perceived self-efficacy for screening and counseling patients is associated with physicians’ counseling of patients with unhealthy alcohol use, and patients’ subsequent drinking.

**Methods:**

This study is a prospective cohort study (nested within a randomized trial) involving 41 primary care physicians and 301 of their patients, all of whom had unhealthy alcohol use. Independent variables were physicians’ attitudes toward unhealthy substance use and self-efficacy for screening and counseling. Outcomes were patients’ reports of physicians’ counseling about unhealthy alcohol use immediately after a physician visit, and patients’ drinking six months later.

**Results:**

Neither physicians’ attitudes nor self-efficacy had any impact on physicians’ counseling, but greater perceived self-efficacy in screening, assessing and intervening with patients was associated with more drinking by patients six months later.

**Conclusions:**

Future research needs to further explore the relationship between physicians’ attitudes towards unhealthy alcohol use, their self-efficacy for screening and counseling and patients’ drinking outcomes, given our unexpected findings.

## Background

Primary care physicians are expected to regularly screen for and counsel their patients on a wide variety of preventable health problems, such as diabetes, hypertension, depression, and unhealthy alcohol use, the spectrum from levels of use that risk consequences through dependence [1]. Unhealthy alcohol use often goes undetected in primary care because clinicians do not ask about it, and patients with unhealthy alcohol use present either asymptomatically, with early stage problems, or with problems that are not recognized as being alcohol-related [2]. Low perceived self-efficacy for discussing difficult health issues with patients is often responsible for physicians’ low rates of screening and counseling for health behaviors, even when physicians view screening to be important [3-5]. Physicians’ attitudes towards patients and counseling for unhealthy alcohol use may also be another barrier. Knowing the factors that are associated with physicians’ screening and counseling behaviors for unhealthy alcohol use, and how these factors facilitate patients’ drinking behavior change, is paramount to improving patient health outcomes and providing quality care for patients with unhealthy alcohol use [6].

Self-efficacy (i.e., perceived behavioral control) and attitudes are important variables in the Theory of Planned Behavior (TPB) [7]. The TPB emphasizes the role of behavioral intentions in predicting behavior. These intentions are influenced by a person’s attitude towards a behavior, their subjective norms (e.g., pressures to perform a certain behavior) and their perceived behavioral control (e.g., self-efficacy). The TPB also stresses that perceived behavioral control, or self-efficacy, may directly predict behavior without the influence of attitudes, norms or intentions [7]. Therefore, one route to predicting physicians’ screening or counseling of patients with unhealthy alcohol use is that physicians’ attitudes towards counseling patients for unhealthy alcohol use *and* physicians’ self-efficacy for treating patients with unhealthy alcohol use both play a role in determining whether or not they counsel patients with unhealthy alcohol use. For example, in one study, 77% of primary care providers reported that it was important or very important to intervene with patients who report unhealthy alcohol use (positive attitude towards intervention/counseling), yet only 21% of physicians felt they could do this effectively (low self-efficacy) [8]. Another possible route is that physicians’ self-efficacy *alone* has a direct effect on whether or not physicians screen and counsel patients. However, most studies which have examined primary care physicians’ self-efficacy have focused on self-efficacy for general communication skills, not specifically screening and counseling about alcohol use [9]. It is not known whether self-efficacy and attitudes both play a role in screening and counseling for unhealthy alcohol use, or whether self-efficacy alone can predict screening and counseling for unhealthy alcohol use—both of which, in turn, can lead to improved patient outcomes.

This paper presents findings from analysis of data collected for The Screening and Intervention in Primary care (SIP) study, a cluster randomized controlled trial (at the physician level) in an urban, academic primary care practice. The trial results have been published elsewhere [10]. Briefly, patients who screened positive for unhealthy alcohol use were assessed prior to physician visits, interviewed immediately after those visits and then re-interviewed six months later. Primary care physicians in the intervention group were provided with their patients’ alcohol screening results, along with decision support strategies on how to counsel (defined broadly as providing advice, having a discussion or referring for further treatment) their patients during the patients’ visit. Physicians in the control group did not receive that information. Results from the SIP study provided evidence that prompting physicians with alcohol screening results and decision support strategies for action could modestly increase discussions about alcohol use and decrease some patients’ alcohol consumption.

In this article, we use the data collected in physician questionnaires completed prior to patient enrollment in the SIP trial on physicians’ attitudes towards treating patients with unhealthy alcohol and drug use and their perceived self-efficacy for screening and counseling patients, to examine whether these factors were associated with physicians’ screening and counseling practices and patients’ drinking outcomes six months later. Not all physicians who received patient screening results and decision support strategies in the main study counseled their patients, and some physicians in the control group of the main study still counseled their patients on unhealthy alcohol use even though they did not receive their patients’ screening results. Thus, the current analyses are focused on determining whether physicians’ self-efficacy and attitudes were associated with patients’ reports of alcohol counseling at the index visit, and patient drinking levels six months later. Using the TPB as our guide, we aimed to examine whether 1) physicians who report positive attitudes towards patients’ alcohol and other drug-related use are more likely report higher perceived self-efficacy for counseling patients, and their patients are more likely to report decreased drinking, and 2) physicians’ who report higher perceived self-efficacy for counseling patients with unhealthy alcohol use will result in patients reported decreased drinking six months later.

## Methods

### Setting

The urban academic primary care practice where this study took place is part of the largest safety-net hospital in the U.S. Northeast. Approximately 73% of the nearly 860,000 outpatient visits in 2012 were from a population who are underserved, low-income, or are elderly.

### Participants

Resident and faculty physicians at this clinic who had seen 80 or more patients in the previous three years and who did not anticipate leaving the practice within six months were recruited, enrolled and randomly assigned to study group before patients were enrolled. Physicians were informed that the investigators would conduct a health screening study.

Patients enrolled in the study were those who consumed alcohol in the past month and were identified as having unhealthy alcohol use [2], defined by 1) answering yes to one or more of the four CAGE alcohol screening questions [11] (modified to refer to the past month) or 2) having consumed hazardous amounts of alcohol in the past month, using the Timeline Followback method (TLFB) [12]. CAGE screening questions are as follows: 1) Have you felt that you should cut down on your drinking? 2) Have people annoyed you by criticizing your drinking? 3) Have you felt bad or guilty about your drinking? 4) Have you had a morning eye-opener? Patients were asked to respond yes or no to each question. Hazardous amounts for men and women, respectively, as assessed by TLFB, were defined as more than 4 standard drinks on an occasion or 14 drinks per week on average, and as more than 3 standard drinks on an occasion or 7 drinks per week on average in the past 30 days. We used a definition of unhealthy alcohol use that spans a spectrum from risky use, through alcohol use disorders and alcohol dependence [2].

### Procedure

Prior to enrolling any patients into the study, physicians completed a confidential written survey, consisting of questions either derived from previous surveys (i.e., physician attitude items [13]) or created for this study (i.e., physician self-efficacy/confidence questions). These survey items assessed physicians’ attitudes towards patients with unhealthy alcohol and drug use and their perceived self-efficacy in counseling patients about unhealthy substance use. A trained staff researcher screened and enrolled patients before their visit with a physician, and again, talked to the patient immediately following the consultation. The assessment that occurred before the physician visit involved drinking amounts [14] and demographics. Immediately after the physician visit, patient interviews determined whether the physician counseled them about their unhealthy alcohol use. Interviews also assessed medical comorbidity, whether they had previously seen the physician. Six months later, patients were interviewed by telephone to determine alcohol consumption in the past 30 days [15]. All patients provided informed consent, and all ethical standards for protecting human subjects were followed in accordance with standards of Boston University’s internal review board for the protection of human subjects and the Helsinki Declaration of 1975. A certificate of confidentiality was obtained from the federal government to further protect participant privacy.

### Independent variables

#### Physicians’ attitudes

Thirteen of the original 50 items of the Substance Abuse Attitude Scale were used to measure physicians’ attitudes towards patients’ alcohol and other drug use [13]. All items were scored on a five point Likert scale ranging from strongly disagree to strongly agree. Negatively worded items were reverse scored. The 13 items were selected for this study because of their perceived importance for a primary care population. Previous principal components analyses showed that these 13 items loaded onto three distinct factors [16]. These factors, or subscales, were labeled ‘Positive treatment beliefs (8 items, Cronbach’s alpha = 0.83)’; ‘Negative attitudes towards patients’ (3 items, Cronbach’s alpha = 0.56); and ‘Addiction as treatable’ (2 items, Cronbach’s alpha =0.96). Examples from each factor are listed below:

*Positive Treatment Beliefs:* “Physicians who diagnose alcoholism early improve treatment success”.

*Negative attitudes towards patients:* “Most alcohol and drug dependent persons are unpleasant to work with as patients”.

*Addiction as treatable*: “Alcoholism is a treatable illness”.

#### Physicians’ self-efficacy

Perceived self-efficacy is defined in the current study as physicians’ confidence in their ability to perform the necessary skills for counseling patients to reduce their alcohol intake. Ten items were created for this study to measure physicians’ perceived self-efficacy in their ability to discuss and communicate information on unhealthy substance use with their patients. These 10 items factor analyzed into 3 distinct subscales: ‘Screening’ (3 items, Cronbach’s alpha = 0.73), ‘Initiating change’ (2 items, Cronbach’s alpha = 0.73), and ‘Assessment and Intervention’ (5 items, Cronbach’s alpha = 0.84). The 10 items were measured on a five-point Likert scale ranging from very confident to not at all confident. Negatively worded items were reverse scored. Examples from each factor are listed below:

*Screening:* “How confident are you in your skills for assessing a patient’s risk for developing problems from alcohol use?”

*Initiating Change:* “How confident are you in your skills for initiating change in patients’ drinking or drug use?”

*Assessment and intervention:* “How confident are you in your skills referring patients for alcohol or drug treatment?”

### Outcomes

#### Physician’s counseling of patients

Immediately after the visit, patients were asked to respond *yes or no* to questions about whether they had received alcohol counseling during that visit, defined as *discussion* about safe drinking limits (one item), *advice* to cut down or abstain from alcohol (four items), or *referral* to an alcohol specialist or treatment program (three items). An example question was: “Did the doctor give you any advice about your drinking habits?” If patients responded “yes” to any of these questions, physicians were considered to have counseled the patient about unhealthy alcohol use. Patient exit interview questions similar to these have been validated in previous studies [12,17].

#### Patient drinking outcomes

We measured two patient drinking outcomes: 1) *drinks per day* at six month follow-up, and 2) *hazardous drinking* levels (as defined above) at the six month follow-up. Both were assessed using the Timeline Followback method (TLFB), which is a retrospective daily calendar method that seeks to obtain day-to-day estimates of drinking for periods of up to one year prior to the administration date [18]. In the current study, participants were asked to complete the TLFB for the previous 30 days. The TLFB is a psychometrically sound drinking assessment method which is designed to capture all drinking, including sporadic heavy days and unpatterned drinking. People are prompted with a calendar on which they write important events that serve as memory prompts for estimating alcohol consumption on each day during the reporting interval. On average, it takes approximately 20–30 minutes to complete a TLFB for a one year interval [18]. The TLFB was administered in the current study in person for the baseline measure of drinks per day, and by telephone at six months.

### Covariates

Important covariates were determined by clinical and demographic importance (patients’ age, education, comorbidities, race, sex and whether or not the patient had ever previously met the doctor). Additionally, we controlled for physician level of training (resident or faculty), whether the physician was in the control or intervention group in the parent study, and for the six month follow-up analyses, we controlled for patients’ baseline drinking.

### Statistical analyses

We first created descriptive statistics of key variables. Generalized estimating equations (GEE) were then used to adjust for clustering of patients by physician (PROC GENMOD, SAS software 9.1) [19]. For continuous outcomes (patients’ drinks per day), we specified the identity link function; for dichotomous outcomes (physicians’ counseling and patients’ hazardous drinking levels), we specified the logit link function. These models adjusted for clustering of patients by physician, with simultaneous adjustment for patient and physician covariates. We specified an exchangeable working correlation structure and empirical variance estimator. We did not adjust for multiple comparisons.

## Results

Forty-one physicians completed the survey prior to the start of the study. Twenty-two physicians were faculty-level and 19 were resident physicians. The average age of the physicians was approximately 34 years (with a range of 26 to 59 years), 56% were male and 65% were white. Of the 301 patients who enrolled in the study at baseline and provided alcohol counseling data immediately after the visit, 70 patients visited resident physicians and 231 patients visited faculty physicians (Table 1). The majority of patients (69%) were seeing physicians they had seen previously. Fifty-six percent of patients were African American, 16% were Latino and 19% were white. Sixty-three percent of patients were male, and 62% had graduated from high school. Seventy-eight percent (n = 235) of patients reported that they received *any* counseling about unhealthy alcohol use from their physician. Six months following the baseline visit, 77% (n = 231) of patients of 35 physicians were successfully contacted and reported on their drinking. The average number of drinks per day was 2.45 at baseline and 2.71 at six months follow-up (Table 1). Of these 231 patients with six month drinking information, 54% (n = 125) reported drinking hazardous amounts of alcohol in the past 30 days.

**Table 1:** Physician and patient demographic data

**Patient characteristics (n = 301 at baseline)**	**Number**	**% or SD**
Male	190	63.1%
Age in years (mean, SD)	42.9	13
Ethnicity		
African American	170	56.5%
Latino	47	15.6%
White	58	19.3%
Other	26	8.6%
Graduated from high school	189	62.8%
Had one or more medical comorbidities	206	68.4%
Has previously met doctor visiting today	209	69.4%
Physician counseled about unhealthy alcohol use	235	78.1%
Drinks per day at baseline (mean, SD)	2.45	4.26
Drinks per day at 6 month follow-up (mean, SD) n = 231	2.71	7.56
**Physician characteristics (n = 41 at baseline)**		
Male	23	56%
Age in years (mean, SD)	34.4	7.04
Ethnicity		
African American	4	9.8%
Latino	2	4.9%
White	27	65.8%
Other	8	19.5%
Faculty level physician	22	53%

**Figure 1:**
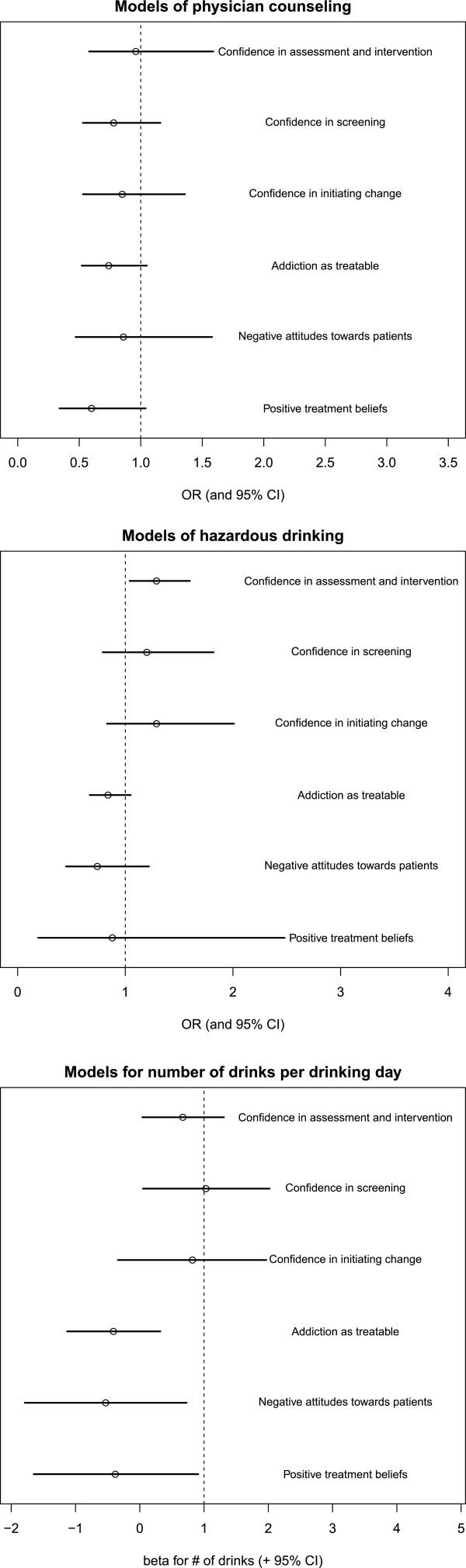
**Association between physicians’ attitudes and perceived self-efficacy (confidence) and patients’ alcohol counseling (baseline) or patients**’ **drinking outcomes (6 month follow-up).**

Although physicians generally reported positive attitudes towards counseling patients about unhealthy substance use, positive treatment beliefs and more positive attitudes towards those with substance use disorders (Table 2), these attitudes were not significantly associated with patients’ reports of whether or not the physician counseled them about unhealthy alcohol use or patients’ drinking outcomes (either drinks per day or hazardous drinking levels) at six months after the physician visit at study entry (Figure 1, Table 3). No statistically significant differences between physicians’ self-efficacy (confidence) and physicians’ attitudes emerged (Table 2). However, some aspects of physicians’ self-efficacy was associated with patients’ drinking outcomes six months later. Higher perceived self-efficacy for screening, and for assessing and intervening with patients, were associated with more drinking in patients six months later (Figure 1, Table 3).

**Table 2:** Physicians’ attitudes and their perceived self efficacy (confidence) for counseling patients (N = 41)

**Attitude subscales**	**Mean**	**SD**
Positive treatment beliefs (8 items)	4.34	0.49
Negative attitudes towards patients (3 items)	4.37	0.45
Addiction as treatable (2 items)	4.21	0.72
**Self-efficacy subscales**	**Mean**	**SD**
Confidence in initiating change (2 items)	3.63	0.54
Confidence in screening (3 items)	4.19	0.48
Confidence in assessment and intervention (5 items)	3.54	0.87

Scales ranged from strongly disagree (1) to strongly agree (5).

**Table 3:** Results of GEE models of association between physicians’ attitudes and perceived self-efficacy (confidence) and patients’ reports of physicians’ alcohol counseling (baseline) or patients’ drinking outcomes (6 month follow-up)

**Item**	**GEE model of physicians’ counseling N = 301 patients**		**GEE model of patients’ hazardous drinking N = 231 patients**		**GEE model of patients’ drinks per day N = 231 patients**	
	**Adjusted OR (95**% **CI)**	***p***	**Adjusted OR (95**% **CI)**	***p***	**Beta (95**% **CI)**	***p***
Positive treatment beliefs	0.60 (0.34, 1.04)	0.07	0.88 (0.19, 2.48)	0.53	−0.38 (−1.65, 0.91)	0.57
Negative attitudes towards patients	0.86 (0.47, 1.58)	0.63	0.74 (0.45, 1.22)	0.24	−0.53 (−1.79, 0.73)	0.41
Addiction as treatable	0.74 (0.52, 1.05)	0.09	0.84 (0.67, 1.05)	0.13	−0.41 (−1.13, 0.32)	0.27
Confidence in initiating change	0.85 (0.53, 1.36)	0.51	1.29 (0.83, 2.01)	0.26	0.82 (−0.34, 1.97)	0.17
Confidence in screening	0.78 (0.53, 1.16)	0.88	1.20 (0.79, 1.82)	0.40	**1.03 (0.05, 2.02)**	**0.04**
Confidence in assessment and intervention	0.96 (0.58, 1.59)	0.23	**1.29 (1.04, 1.60)**	**0.02**	**0.67 (0.04, 1.31)**	**0.04**

GEE: Generalized estimating equation; CI: Confidence interval; Covariates in each model: physician level (attending or resident), physician randomization group (to receive screening results or not), patient race/ethnicity (African American, White, Latino, Other), gender, high school graduate, patient met doctor before, any patient medical comorbidity.

## Discussion

Our study is one of few to have examined the effect of social psychological constructs on reports of physicians’ counseling of patients with unhealthy alcohol use and patients’ reported drinking outcomes in a real-world, urban setting. We found a significant effect of physicians’ perceived self-efficacy—an important factor in the Theory of Planned Behavior--on patient drinking levels. Greater physician self-efficacy was associated with more patient drinking, a finding that was in the opposite direction from that hypothesized. It is conceivable that greater self-efficacy could be associated with worse clinical outcome, if physicians were overly confident, or if their confidence was not at all associated with or perhaps negatively correlated with their actual clinical skill. Our findings should be viewed as cautionary until further replication.

A strength of this study is that it was not an assessment of how physicians or patients might behave based on vignettes or hypothetical situations. This study involved patients who, in fact, had unhealthy alcohol use and their actual, clinic-based physicians. Most patients in our study (69%) had previously visited with the physician in the study. A recent systematic review of 36 communication interventions studies found that only three were focused on assessments involving both the patient and the physician [20]. Most interventions with patients were carried out in clinic waiting rooms, not during the actual consultation with their physicians, and the interventions that involved physicians consisted of role-play and feedback sessions, not actual consultations with patients where real communication could take place. Our study is therefore one of the few studies that involves patients and physicians in clinical practice who were previously known to each other. One potential reason for our unexpected findings may be related to this real-world population. It is possible that one or more conversations with patients about unhealthy alcohol use is not enough to warrant long-term change. It is also possible that physicians who perceived themselves as confident to discuss unhealthy alcohol use with their patients were not able to create meaningful change, either through the words that they used during the consultation with the patient, or because the patient decided not to, or was not able to, heed this advice. Previous research has found that providers focus their advice to abstain on patients with the most severe drinking problems, a population whose behavior is unlikely to change [21]. Moreover, clinic staff members’ perceptions of their personal efficacy, organizational factors involved in implementation of a screening program, and not only physicians’ self-efficacy, is important for engaging patients in treatment for unhealthy alcohol use [22,23].

Studies that have reported significant associations between physicians’ positive attitudes and their counseling of patients have assessed attitudes and counseling behavior simultaneously, at the conclusion of an intervention [24], or have reported on the link between attitudes and intended behavior, not actual behavior [25]. In studies where greater physician self-efficacy was associated with improved counseling about patients’ health behaviors, this assessment of self-efficacy was done in the context of interventions where physicians were taught specific clinical skills in order to carry out such counseling [26,27]. In the current study, where physicians’ attitudes were assessed prior to enrolling patients, and physician’ self-efficacy was measured in the absence of any specific clinical skill training, no association was found between attitudes, self-efficacy and physicians’ counseling about unhealthy alcohol use. Thus, it is possible that study designs and specific skills training in previous studies may have been partially responsible for the positive association between attitudes, self-efficacy and counseling behavior. Again, our results showing increased patient drinking associated with increased phyisician self-efficacy for counseling require replicating before further action is taken.

Many studies which have shown positive and significant effects of physicians’ counseling about unhealthy alcohol use on their patients’ drinking outcomes have also involved physicians who had undergone training for this counseling [28,29]. Our study involved physicians who had consented to participate in the study but who did not receive training for screening, counseling or assessing patients who are problem drinkers, a population that represents many primary care providers who are often presented with their patients’ unhealthy behaviors without advance knowledge or training of how to effectively treat their patients. The quality of care for patients with unhealthy alcohol use has been documented as being among the lowest quality in the U.S., compared to other health conditions [30]. Brief counseling for unhealthy alcohol can enhance the quality of primary care through improved communication with patients, greater trust in physicians and physicians’ greater knowledge of their patients’ health concerns, values and beliefs [2]. Therefore, even positive attitudes and greater self-confidence are likely insufficient without specific clinical skills. Primary care physicians need to receive specific training for brief counseling of patients with unhealthy alcohol use in order to effectively treat patients who present with these problems [31].

A limitation of this study was that conversations between physicians and patients were not audio recorded and that self-reports of counseling were not corroborated through these recordings. It is possible that physicians did counsel patients about their unhealthy alcohol use (involving different aspects of counseling such as advice, discussion or referral), but that patients did not perceive it as alcohol counseling. Evidence for physicians’ counseling of patients about unhealthy alcohol use came only from patients’ reports. In previous studies relying only on self-reports, investigators have successfully validated patients’ reports of physicians’ communication of advice or counseling on health behaviors through similarly designed exit interviews [12,17]. Future research should consider an objective measure of counseling, such as a digital audio recorder that can be turned on and off by the physician or a research assistant at the start and stop of each patient consultation. However, such studies could have different limitations, such as the possible effect of recording on what is said during a visit. Currently, self-report is the best way to assess self-efficacy, and is also the best way to assess hazardous drinking amounts at the levels of interest in this study since laboratory testing detects only much higher amounts. Furthermore, self-reports were obtained with assurances of confidentiality by research staff not involved in the patient’s care, and it seems unlikely that drinking amounts would be differentially reported by patients in relation to physician self-efficacy. A final limitation of this study is that we did not correct for multiple comparisons of the data.

United States Preventive Services Task Force guidelines for screening for unhealthy alcohol use instruct physicians to counsel patients about their alcohol use following a positive, high risk screen [1,32]. This counseling is less structured than a screening test, and is up to the physician to determine what components should be covered—such as advice to limit drinking or cut back entirely, discussion involving the provision of information through leaflets, or referrals to alcohol treatment centers. Seminal work in the field of attitudes and persuasion demonstrated that *how* a message is communicated is as important as the communicator and the message itself [33]. It is possible that physicians who counseled patients were physicians who were confident in communicating sensitive information. However, unless consultations are audio-recorded, the impact of the confidence with which a physician communicates, and the message of counseling itself on patients’ behavior change, may not be known. Further work is needed to determine the mechanisms of change in patients’ drinking behavior, and studies need to be designed to corroborate self-reported behavior with more objective measures, and to ensure that sources of bias can be determined.

## Conclusion

In summary, in this sizeable prospective study of physicians and their patients with unhealthy alcohol use, physician self-efficacy, which is thought to be associated with clinical behaviors, was related to patient drinking outcomes, but in an unexpected direction. Self-efficacy and attitudes may still be important, but not sufficient to affect physicians’ practices.

## Competing interests

Dr. Saitz is employed by Boston University as a clinician, researcher and educator focused on addressing the identification and management of unhealthy substance use. He is and has been the principal investigator for government-funded grants to Boston Medical Center on this topic. He also regularly consults to other government grant-funded projects, as an expert reviewer of legal cases, and as a speaker at academic institutions and professional conferences on these topics.

## Authors’ contributions

ARE developed the research question, contributed to the analysis and interpretation of the data and wrote and edited the manuscript. NH analyzed and interpreted the data and edited the manuscript. RS designed the original study, oversaw the data collection, contributed to the analysis and interpretation of the data, and edited the manuscript. All authors read and approved the final manuscript.

## References

[B1] Whitlock EP Polen MR Green CA Orleans T Klein J Behavioral counseling interventions in primary care to reduce risky/harmful alcohol use by adults: a summary of the evidence for the U.S. Preventive services task force Ann Intern Med 2004 140 557 568 10.7326/0003-4819-140-7-200404060-00017 15068985

[B2] Saitz R Unhealthy alcohol use New Engl J Med 2005 352 596 607 10.1056/NEJMcp042262 15703424

[B3] Mirand AL Beehler GP Kuo CL Mahoney MC Physician perceptions of primary prevention: qualitative base for the conceptual shaping of a practice intervention tool BMC Public Health 2002 2 16 22 10.1186/1471-2458-2-16 12204096 PMC126210

[B4] Gramling R Nash J Siren K Eaton C Culpepper L Family physician self-efficacy with screening for inherited cancer risk Ann Fam Med 2004 2 130 132 10.1370/afm.60 15083852 PMC1466652

[B5] Yoast RA Wilford BB Hayashi SW Encouraging physicians to screen for and intervene in substance use disorders: obstacles and strategies for change J Addictive Dis 2008 27 77 97 10.1080/10550880802122687 18956531

[B6] Saitz R Horton NJ Cheng DM Samet JH Alcohol counseling reflects higher quality of primary care J Gen Intern Med 2008 23 1482 1486 10.1007/s11606-008-0574-4 18618204 PMC2518021

[B7] Ajzen I The theory of planned behavior Organization Behav Human Dec Process 1991 50 179 211 10.1016/0749-5978(91)90020-T

[B8] Kaner E Lock C McAvoy B Heather N Gilvarry E A RCT of three training and support strategies to encourage implementation of screening and brief alcohol intervention by general practitioners Brit J Gen Pract 1999 49 699 703 10756610 PMC1313496

[B9] Gulbrandsen P Jensen BF Finset A Blanch-Hartigan D Long-term effect of communication training on the relationship between physicians’ self-efficacy and performance Patient Educ Couns 2013 91 180 185 10.1016/j.pec.2012.11.015 23414658 PMC3622152

[B10] Saitz R Horton NJ Sullivan LM Moskowitz MA Samet JH Addressing alcohol problems in primary care: a cluster randomized, controlled trial of a systems intervention. The screening and intervention in primary care (SIP) study Ann Intern Med 2003 138 372 382 10.7326/0003-4819-138-5-200303040-00006 12614089

[B11] Mayfield D McLeod G Hall P The CAGE questionnaire: validation of a new alcoholism screening instrument Am J Psych 1974 131 1121 1123 10.1176/ajp.131.10.1121 4416585

[B12] Adams A Ockene JK Wheeler EV Hurley TG Alcohol counseling: physicians will do it J Gen Intern Med 1998 13 692 698 10.1046/j.1525-1497.1998.00206.x 9798817 PMC1500899

[B13] Chappel JN Veach TL Krug RS The substance abuse attitude survey: an instrument for measuring attitudes J Stud Alcohol 1985 46 48 52 3974235 10.15288/jsa.1985.46.48

[B14] Sobell LC Sobell MB Alcohol timeline followback users’ manual 1995 Toronto, Canada: Addiction Research Foundation

[B15] Sobell LC Brown J Leo GI Sobell MB The reliability of the alcohol timeline followback when administered by telephone and by computer Drug Alcohol Depend 1996 42 49 54 10.1016/0376-8716(96)01263-X 8889403

[B16] Saitz R Friedmann PD Sullivan LM Winter MR Lloyd-Travaglini C Moskowitz MA Samet JH Professional satisfaction experienced when caring for substance-abusing patients J Gen Intern Med 2002 17 373 376 12047735 10.1046/j.1525-1497.2002.10520.x PMC1495049

[B17] Pbert L Adams A Quirk M Hebert JR Ockene JK Luippold RS The patient exit interview as an assessment of physician-delivered smoking intervention: a validation study Health Psych 1999 18 183 188 10.1037//0278-6133.18.2.183 10194054

[B18] Sobell LC Sobell MB Connors GJ Agrawal S Assessing drinking outcomes in alcohol treatment efficacy studies: selecting a yardstick of success Alcohol Clin Exper Res 2003 27 1661 1666 10.1097/01.ALC.0000091227.26627.75 14574238

[B19] Liang KY Zeger SL Longitudinal data analysis using generalized linear models Biometrika 1986 173 13 22

[B20] Rao JK Anderson LA Inui TS Frankel RM Communication interventions make a difference in conversations between physicians and patients: a systematic review of the evidence Med Care 2007 45 340 349 10.1097/01.mlr.0000254516.04961.d5 17496718

[B21] Burman ML Kivlahan D Buchbinder M Broglio K Zhou SH Merrill JO McDonnell MB Fihn SD Bradley KA for the Ambulatory Care Quality Improvement Project (ACQUIP) Investigators J Subst Abuse Dis 2004 65 621 630

[B22] Broome KM Flynn PM Knight DK Simpson DD Program structure, staff perceptions and client engagement in treatment J Subst Abuse Treat 2007 23 149 158 17434709 10.1016/j.jsat.2006.12.030 PMC2140244

[B23] van Beurden I Anderson P Akkermans RP Grol RPTM Wensing M Laurant MGH Involvement of general practitioners in managing alcohol problems: a randomized controlled trial of a tailored improvement programme Addiction 2012 107 1601 1611 10.1111/j.1360-0443.2012.03868.x 22372573

[B24] Meredith LS Yano EM Hickey SC Sherman SE Primary care provider attitudes are associated with smoking cessation counseling and referral Med Care 2005 43 929 934 10.1097/01.mlr.0000173566.01877.ac 16116358

[B25] Vogt F Hall S Marteau TM General practitioners’ beliefs about effectiveness and intentions to recommend smoking cessation services: qualitative and quantitative studies BMC Fam Pract 2007 8 39 10.1186/1471-2296-8-39 17615061 PMC1934905

[B26] Lustig JL Ozer EM Adams SH Wibbelsman CJ Fuster CD Bonar RW Irwin CE Jr Improving the delivery of adolescent clinical preventive services through skills-based training Pediatrics 2001 107 1100 1107 10.1542/peds.107.5.1100 11331693

[B27] Seale JP Shellenberger S Boltri JM Okosun IS Barton B Effects of screening and brief intervention training on resident and faculty alcohol intervention behaviours: a pre-post intervention assessment BMC Fam Pract 2005 6 46 10.1186/1471-2296-6-46 16271146 PMC1310533

[B28] Fleming MF Mundt MP French MT Manwell LB Stauffacher EA Barry KL Brief physician advice for problem drinkers: long-term efficacy and benefit-cost analysis Alcohol Clin Exps Res 2002 26 36 43 10.1111/j.1530-0277.2002.tb02429.x 11821652

[B29] Ockene JK Reed GW Reiff-Hekking S Brief patient-centered clinician-delivered counseling for high-risk drinking: 4-year results Ann Behav Med 2009 37 335 342 10.1007/s12160-009-9108-5 19707840

[B30] McGlynn EA Asch SM Adams J Keesey J Hicks J DeCristofaro A Kerr EA The quality of health care delivered to adults in the United States New Engl J Med 2003 348 2635 2645 10.1056/NEJMsa022615 12826639

[B31] Cabana MD Rand C Slish K Nan B Davis MM Clark N Pediatrician self-efficacy for counseling parents of asthmatic children to quit smoking Pediatrics 2004 113 78 81 10.1542/peds.113.1.78 14702452

[B32] Jonas DE Garbutt JC Amick HR Behavioral counseling after screening for alcohol misuse in primary care: a systematic review and meta-analysis for the U.S. Preventive services task force Ann Intern Med 2012 157 645 654 10.7326/0003-4819-157-9-201211060-00544 23007881

[B33] Hovland CI Janis IL Kelley HH Communication and persuasion: psychological studies of opinion change 1953 New Haven: Yale University Press

